# Nervous Diseases and Their Diagnosis

**Published:** 1887-11

**Authors:** 


**Nervous Diseases and their Diagnosis. A Treatment upon the Phenomena Produced by Diseases of the Nervous System, with Especial Reference to the Recognition of their Causes.** By H. C. Wood, M. D., L.L. D., member of the National Academy of Science. Philadelphia: J. B. Lippincott Company. 1887.

The extensive experience which Dr. H. C. Wood has had in the nervous clinics of Philadelphia, pre-eminently fits him for the work he has undertaken. The introduction, which is a general discussion of disease and of neurasthenia, is very interesting and instructive. The next subject, that of paralysis, detection of paralysis, functional palsies, organic palsies, etc., is a very exhaustive and yet concise treatise on this subject. Sixty pages are devoted to it. The following chapters on " Motor Excitements," " Reflexes," Disturbances of Equilibration," "Trophic Lesions," " Sensory Paralysis," "Exaltations of Sensibility," " Disturbances of the Special Senses," " Disorders of Memory and Consciousness," " Disorders of Consciousness," and " Disturbances of Intellection," are equally well written. As will be seen, the book takes up subjects little understood by the profession. They are treated in such a manner as to prove interesting reading. This work fills a long-felt need in this department.

